# The Role of Relational Entitlement, Self-Disclosure and Perceived Partner Responsiveness in Predicting Couple Satisfaction: A Daily-Diary Study

**DOI:** 10.3389/fpsyg.2021.609232

**Published:** 2021-03-23

**Authors:** Octav Sorin Candel, Maria Nicoleta Turliuc

**Affiliations:** Department of Psychology, Faculty of Psychology and Educational Sciences, Alexandru Ioan Cuza University of Iasi, Iasi, Romania

**Keywords:** entitlement, couple satisfaction, self-disclosure, perceived partner responsiveness, daily diary

## Abstract

Recent research has investigated how the sense of relational entitlement (SRE, the extent to which a person expects that his/her needs and wishes will be fulfilled by the romantic partner) diminishes couple satisfaction, but little is known about how SRE affects the daily quality of close, romantic relationships. Moreover, the evidence on how SRE interacts with other features of a satisfying relationship (such as the variables of the interpersonal process model of relationships—self-disclosure, perceived partner disclosure, and perceived partner responsiveness) is scarce. Using an electronic daily diary, we examined 99 couples (198 participants) for 7 days, with two daily measurements for each partner. We used a dyadic double intercept multilevel model, which simultaneously computes effects for men and women. We tested a model where one partner's daily couple satisfaction was predicted by their overall levels of SRE (excessive, restricted, and assertive) and by their daily and overall levels of self-disclosure, perceived partner self-disclosure, and perceived partner responsiveness. The model also included person-level interactions and cross-level interactions between the SRE types and variables of the interpersonal process model of relationships for each gender. The analysis indicated that person-level excessive SRE lowers couple satisfaction. Also, day and person-level perceived partner responsiveness and person-level self-disclosure are related to couple satisfaction, but the latter association is significant only for men. Finally, we found some significant person-level interactions that account for changes in couple satisfaction. For men, the links between couple satisfaction, excessive and restricted SRE were moderated by self-disclosure and perceived partner responsiveness, respectively, perceived partner self-disclosure and perceived partner responsiveness. For women, the associations between couple satisfaction, restricted and assertive SRE were moderated by self-disclosure, respectively, perceived partner self-disclosure. This study advances our understanding of the general implications of SRE in the dynamics of couple relationships. More specifically, it shows how SRE interacts with other couple-specific variables in shaping day-to-day couple satisfaction. The theoretical and clinical implications for couple therapy are discussed.

## Introduction

Inside their romantic relationships, people express and fulfill some of the most intimate needs. Hence, intimate relationships become a crucial context where entitlement-related behaviors take shape (George-Levi et al., 2014). Although generally considered a negative personality trait (Campbell et al., 2004; Grubbs and Exline, 2016) or a facet of narcissism (Miller et al., 2012), some scholars indicate that entitlement also has some adaptive characteristics (Levin, 1970; Moses and Moses-Hrushovski, 1990). Entitlement refers to an outcome that individuals believe they deserve to receive from their relationships (Attridge and Berscheid, 1994). This outcome is important because it allows the distribution of resources within romantic relationships (Lerner and Mikula, 1994). Moreover, other scholars refer to the entitlement as a crystallization of early attachment bonds (Tolmacz, 2011). Thus, just as attachment can be secure or insecure, entitlement may be adaptive or maladaptive.

Previous research showed that the maladaptive forms of entitlement are detrimental to couple satisfaction (Tolmacz and Mikulincer, 2011; George-Levi et al., 2014), but the more adaptive forms were not related to couple satisfaction (George-Levi et al., 2014). However, no study, to our knowledge, explored whether these relationships are influenced by other variables. For example, previous studies have shown that the expression of needs and the partner's responsiveness to those needs shape couple satisfaction (Bar-Kalifa et al., 2015; Unger et al., 2015). For this reason, the main goal of this study was to explore the moderating role of self-disclosure, perceived partner disclosure, and perceived partner responsiveness (PPR) in the relationship between sense of relational entitlement (SRE) and couple satisfaction. To do so, we used a dyadic daily-diary design that allowed us to examine whether and how the daily fluctuations in self-disclosure, perceived partner disclosure, and PPR, and the person level of these variables moderated the association between SRE and couple satisfaction.

## The Sense of Relational Entitlement

The first conceptualization of entitlement most likely belongs to Freud (George-Levi et al., 2014). If Freud (1916) talked about the patients who claimed more compensation for their congenital deficiencies, Jacobson (1959) suggested that some people may think they deserve more because of the exceptional qualities they believe they have. Later, the concept has been included among the five factors of narcissism, indicating the tendency to expect favored treatment from others (Exline et al., 2004). It is well-documented that narcissism has a negative impact on couple relationships by increasing vengefulness (Brown, 2004), interpersonal aggression (Reidy et al., 2010), and vindictive behavior (Ogrodniczuk et al., 2009). Also, some studies indicate that narcissism may predict higher marital satisfaction and commitment, but only in cases of narcissistic individuals with high self-esteem (Sedikides et al., 2004) and with communal feelings for the partner (Finkel et al., 2009). However, narcissism and entitlement are distinct constructs (Brown et al., 2009). First, more recent research showed a clear distinction between two forms of excessive entitlement, grandiose and vulnerable, both of them being unrelated to narcissism (Crowe et al., 2016). Second, entitlement and narcissism show different relationships with other psychological constructs. For example, while grandiose narcissism is negatively associated with short-term psychological distress, anxiety and depression, entitlement shows no relationships with them (Brown et al., 2009). Third, narcissism is a purely intrapersonal construct, while entitlement is a more interpersonal one (Williams et al., 2018). Finally, narcissism can be conceptualized as a *personality trait* and a *personality disorder* (Lamkin et al., 2017), while entitlement is a trait-like characteristic that can take both adaptive (assertive) and pathological (restricted or inflated) forms.

Although the concept of entitlement was initially described as a negative individual characteristic, researchers understood that people can have a healthy assertion of their need and wishes (Levin, 1970; Kriegman, 1983; Moses and Moses-Hrushovski, 1990). The concept of *sense of entitlement* assumed and promoted this positive dimension. Thus, this concept integrated three basic entitlement-related attitudes: excessive, restricted, and assertive entitlement (Levin, 1970; Kriegman, 1983; Moses and Moses-Hrushovski, 1990). The authors suggest that an *excessive sense of entitlement* characterizes people who believe that their need must be fulfilled regardless of the needs or emotional states of those around. A *restricted sense of entitlement* is present in people characterized by unassertiveness, timidity, which are less independent and less self-assured. Finally, people who are characterized by *assertive sense of entitlement* can realistically estimate what they can expect from others, are assertive and confident that they can achieve their needs and rights. It is an adaptive form of entitlement, essential for the well-being of individuals (George-Levi et al., 2014). As we can see, unlike narcissistic entitlement, the sense of entitlement has both negative and positive dimensions and implications. Moreover, several clinical reports that underlined the important role of the sense of entitlement is couple relationships (e.g., Blechner, 1987; Billow, 1999).

In this context, Tolmacz and Mikulincer (2011) proposed another development of the concept, the *sense of relational entitlement (SRE)*, in order to explain individual differences in expression of need and rights inside dyadic relationships. The authors defined the concept as the extent to which a person expects that his/her needs and wishes will be fulfilled by the romantic partner, and as a person's affective and cognitive responses to a romantic partner's failure to fulfill these needs and hopes (Tolmacz and Mikulincer, 2011). In recent years, the concept of SRE was applied in various studies concerning interpersonal relationships, being linked to caregiving style (George-Levi et al., 2016), attachment orientations (Shadach et al., 2017; Brenner et al., 2019), pathological concern (Shavit and Tolmacz, 2014), dating abuse (Warrener and Tasso, 2017), relationship with parents (Tolmacz et al., 2016), and the quality of one's intimate relationship (Bar-Kalifa et al., 2016; Tolmacz et al., 2017; Williams et al., 2018; Candel and Turliuc, 2019; Turliuc and Candel, 2019). The conceptual innovation of Tolmacz and Mikulincer (2011) was completed with the development of a specific scale for measuring the sense of entitlement in couple relationships: The Sense of Relational Entitlement Scale (SRES). The scale includes subscales for the dimensions of entitlement (assertive, restricted, and excessive), this time in the context or the couple relationship. In other words, the authors indicated that people may be characterized by an assertive (confidence in the relationship and the ability to ask for their rights), restricted (a lack of assertiveness and deservingness), and excessive sense of relational entitlement (negative evaluations of the partner and exaggerated expectations). It seems that people with excessive SRE are more sensitive to negative aspects of the partner and relationship and have higher expectations for their partner attention and understanding (Tolmacz and Mikulincer, 2011). Also, an inflated sense of entitlement has been associated with inadequate early parental care, maldaptive attachment styles, and early trauma, such as sexual abuse (Shadach et al., 2017; Brenner et al., 2019). Thus, it was presumed that this type of relational entitlement will have the highest impact on couple satisfaction because excessively entitled people have stronger reaction to the degree of fulfillment of their needs and wishes (Bar-Kalifa et al., 2016). According to Tolmacz and Mikulincer (2011), two or more types of entitlement (for example, assertive and exaggerated) can coexist in the same individual. Given that this concept measures a type of dispositional entitlement and the fact that a relationship consists of hundreds of daily interactions, Tolmacz and Mikulincer (2011) suggest that a person can have high scores on more than one dimension of entitlement. This reflects the various interactions between partners, some of which are more assertive and adaptive, and others more exaggerated or maladaptive.

## The Sense of Relational Entitlement and Couple Satisfaction

Couple satisfaction represents the “people's global subjective evaluation of the quality of their marriage” (Li and Fung, 2011, p. 246). It can vary as a function of different interpersonal and intrapersonal characteristics of the couple, such as the partner's background and traits (Bradbury et al., 2000). Studies assessing the sense of relational entitlement and its link with couple satisfaction are scarce. As the Sense of Relational Entitlement Scale is being developed and validated only recently, the situation is to be expected. In their study in which they present the construction of the SRE scale, Tolmacz and Mikulincer (2011) presented the associations of SRE with couple satisfaction among young adults (Tolmacz and Mikulincer, 2011). These findings also received support when examining middle-aged long-term dyadic relationships (George-Levi et al., 2014). People with an inflated sense of relational entitlement are more sensitive to the partner's transgressions. This leads them to use more negative tactics in conflict resolution, such as more verbal aggression, more dominance, and less compromise (Williams et al., 2018). Moreover, excessive relational entitlement was strongly related to abusive behaviors in couple relationships (Wood, 2004; Warrener and Tasso, 2017). Other research showed that it was related to divorce rates (Sanchez and Gager, 2000), selfishness in romantic relationships (Exline et al., 2004) and chronic relationship conflict over a period of 10 weeks (Moeller et al., 2009). On the contrary, individuals with a restricted sense of entitlement are more avoidant when resolving conflicts and suffer from more pathological concern (Shavit and Tolmacz, 2014; Williams et al., 2018). Previous studies showed that both excessive and restricted SRE were strong predictors of lower relational satisfaction (Tolmacz and Mikulincer, 2011; George-Levi et al., 2014; Candel and Turliuc, 2019). Having an assertive sense of relational entitlement was suggested to be linked with positive outcomes, such as higher life satisfaction, more self-esteem and self-efficacy (Tolmacz et al., 2016). However, previous studies that tested it in the context of romantic relationships found no link between assertive SRE and satisfaction (Tolmacz and Mikulincer, 2011; Candel and Turliuc, 2019). Thus, previous research points out that SRE is a trait-like psychological characteristic that has a negative impact on satisfaction. In this study, we were interested in exploring whether the between-person differences in SRE (excessive, restricted, and assertive) will lead to different levels of daily couple satisfaction. We hypothesized that:

(H1) Excessive and restricted relational entitlement will be negatively associated with couple satisfaction. Given that assertive entitlement was not related to relational satisfaction, as previous studies have concluded (e.g., Tolmacz and Mikulincer, 2011), we did not make any hypothesis for its associations with couple satisfaction, but it was included in all the analyses.

## Perceived Partner Responsiveness, Self-Disclosure, and Couple Satisfaction

Romantic intimacy is one of the strongest positive predictors of physical health (e.g., lower illness rates, better recovery rates etc.; Hook et al., 2003), psychological well-being (e.g., lower risk for depression, higher levels of life satisfaction; Hook et al., 2003), and couple satisfaction (Dandurand and Lafontaine, 2013; Bar-Kalifa et al., 2015). The Interpersonal Process Model of Intimacy (Reis and Shaver, 1988) indicates that intimacy is built through two fundamental processes: self-disclosure and empathic response from the partner. The model suggests that the ability of both partners to communicate essential information about their wishes, needs, or expectancies, and the perception that a partner is responsive to one's needs is a central construct when it comes to determining the quality of a relationship (Reis et al., 2004). According to this model, when the expression of needs and the response toward those needs are higher, people perceive their romantic relationships as being more intimate (Reis and Shaver, 1988).

Self-disclosure (namely, the verbal communication of information about the self, including personal thoughts, states, dispositions, needs, events in the past, and plans for the future) is a central concept in the study of romantic relationships (Finkenauer et al., 2004). It can be used to maintain proximity to the partner (Lee and Pistole, 2012), it leads to greater intimacy (Laurenceau et al., 1998), and it is generally associated with positive couple outcomes over extended periods of time (Sprecher and Hendrick, 2004). Also, due to the capitalization of these positive outcomes, people who self-disclose more feel an improvement in other aspects of their romantic relationships (Langston, 1994). Previous studies offer evidence that greater self-disclosure is associated with greater couple satisfaction (Hendrick, 1981; Sprecher and Hendrick, 2004; Unger et al., 2015). This may be, in part, due to the role that self-disclosure has in relationship maintenance (Sprecher and Hendrick, 2004), and to its contribution to greater intimacy, which is an important indicator for relational success (Reis and Shaver, 1988). In addition to one's own self-disclosure, perceived partner disclosure might also play a role in the level of satisfaction a person feels. First, one's higher self-disclosure may lead to higher levels of partner disclosure (Laurenceau et al., 2005). Second, Rosenfeld and Bowen (1991) found that the partner's level of disclosure is important for one's couple satisfaction, but people usually overestimate the partner's disclosure. Thus, the perception overcomes the reality of the degree of partner's self-disclosure. Finally, giving and receiving self-disclosure are associated with love, commitment, and couple satisfaction, meaning that these processes sustain the desire to continue the relationship (Sprecher and Hendrick, 2004).

Perceived partner responsiveness (PPR), described as the perception that a partner understands, values, and responds supportively to one's needs, is a cardinal process in the study of relational quality (Reis et al., 2004). When people feel that their partners are more responsive, they believe their relationship is more intimate and that it offers more satisfaction (Laurenceau et al., 1998; Canevello and Crocker, 2010). It has been shown that PPR can mediate or moderate the relationship between various behaviors or traits (e.g., sexual behavior, attachment, or social anxiety) and couple satisfaction (Kane et al., 2007; Bar-Kalifa et al., 2015; Gadassi et al., 2016), and that it can influence variables such as investment, alternatives, or commitment for the relationship (Segal and Fraley, 2016). Moreover, the temporal link between PPR and couple satisfaction was previously validated in multiple studies that employed longitudinal or dyadic diary analysis (Bar-Kalifa et al., 2015; Gadassi et al., 2016; Segal and Fraley, 2016).

Expressing their needs (self-disclosure) and perceiving the partner's response to those needs (PPR) represent organizing constructs that change the way one feels in their relationship. Previous reports showed that perception of enthusiastic, engaged responses from one's partner was associated with more couple satisfaction (see Reis, 2014 for a review). Also, self-disclosure can promote couple satisfaction and endurance (see Finkenauer et al., 2018 for a review). Moreover, past daily diary studies showed that disclosing and receiving disclosure and responsiveness from the partner on a day-to-day basis represent central components of a well-functioning intimate relationship (Laurenceau et al., 2005). Based on these prior findings, we may consider that both the general levels of self-disclosure, perceived partner self-disclosure, and PPR, and the day-to-day expressions of these functional behaviors would positively impact couple satisfaction. Thus, we hypothesized that:

(2) Self-disclosure will be positively associated with couple satisfaction on person-level and day-level. (3) The perceived self-disclosure of the partner will be positively associated with couple satisfaction on person-level and day-level. (4) PPR will be positively associated with couple satisfaction on person-level and day-level.

## The Moderation Role of Self-Disclosure and Perceived Partner Responsiveness

While SRE refers to the extent a person expects his/her needs and wishes will be fulfilled inside a romantic relationship, self-disclosure includes the verbal communication of the person's needs, and perceived partner's responsiveness represents the perception that a partner understands and responds supportively to the person's needs. It is important to note that all these aspects gravitate around need fulfillment in the romantic relationship, being relevant for the person's perception of coupe satisfaction (Patrick et al., 2007). Following the previously mentioned works, we consider that PPR and self-disclosure would change the nature (would be a moderator) of the associations between SRE and couple satisfaction by diminishing the strength of the previously found negative associations.

First, both PPR and self-disclosure foster positive outcomes that may influence the levels of couple satisfaction. For example, daily PPR encourages individuals to express more joy, excitement, contentment, and gratitude (Ruan et al., 2020). Also, PPR was positively related to forgiveness after a real live hurtful event (Pansera and la Guardia, 2011). In regard to self-disclosure, the partners who are more involved in such behaviors experience greater emotional involvement, greater satisfaction, and positive affect after being taking part in couple conflicts (Prager et al., 2015).

Second, we found evidence that SRE is associated with relationship quality variables (Tolmacz and Mikulincer, 2011; George-Levi et al., 2014; Candel and Turliuc, 2019). While the expression of needs is crucial in one's relationships, the inter-individual differences can make the most important difference in how people present their needs in their romantic relationships (Clark et al., 2001). Coming from a person's earlier experiences with fulfillment needs (Tolmacz, 2011), SRE plays a relevant role in an individual's general expectation toward a relationship and in the outcome of the said relationship. However, previous studies have shown that positive intimate experiences can counter a person's maladaptive expectation from a relationship (Stanton et al., 2017). For the individuals with an avoidant attachment style, engaging in intimacy-promoting behaviors led to a higher relational quality immediately after engaging in the said behaviors (Stanton et al., 2017). Experiences with people who are understanding, trustworthy, and responsive to one's needs will lead to positive views of others, whereas relationships with people who are unresponsive and rejecting will lead to negative views of others (Bretherton, 1990; Grabill and Kerns, 2000).

Third, there is evidence for the moderating role of the intimacy-related variables on the association between various personal or couple constructs and relationship quality. For example, self-disclosure can soften the harmful effects of negative interactions on need fulfillment and can alleviate the negative effects of trauma on satisfaction (Prager and Buhrmester, 1998; Monk and Nelson Goff, 2014). A study assessing the moderation role of PPR showed that at higher levels of PPR, more self-focused talk was associated with higher sexual satisfaction, and at lower levels of PPR, more self-focused talk was associated with lower sexual satisfaction (Merwin and Rosen, 2020). Also, emotional intimacy was found to moderate the relationship between the use of Sexually Explicit Media (SEM) and relationship satisfaction in men's case, with a higher SEM significantly associated with lower relationship satisfaction among men reporting lower levels of emotional intimacy (Veit et al., 2017).

Based on this evidence, it is reasonable to expect that the impact of adaptive or maladaptive types of SRE on couple satisfaction may vary as a function of self-disclosure and PPR.

Moreover, with evidence coming from both cross-sectional (Kane et al., 2007; Unger et al., 2015) and daily diary studies (Bar-Kalifa et al., 2015; Gadassi et al., 2016; Segal and Fraley, 2016), we consider that both the person-level and day-level effects should be taken into account. We consider that having a relationship characterized by positive and intimacy-promoting behaviors such as PPR, self-disclosure, and perceived partner self-disclosure (in general and on a day-to-day basis) would buffer the negative effects of SRE on relational satisfaction. Individuals with excessive or restricted SRE feel more negativity and, in the case of the former, are more conflictual in nature. This can be counteracted by positive partner behaviors (PPR and perceived partner self-disclosure) and by more intimacy-promoting behavior on their part (self-disclosure). Thus, we hypothesized that:

(5) The association between SRE (exaggerated, restricted, or assertive), on the one hand, and couple satisfaction, on the other hand, will be weaker for those characterized by high levels of self-disclosure, perceived partner self-disclosure or PPR.

Finally, although we did not propose any hypothesis for it, we explored the role of gender in shaping these relationships.

## Method

### Participants

Ninety-nine couples (198 participants) responded to a 7-day, two measurements each day dyadic diary. For men, the mean age was 25.74 years (SD = 5.63, Min. = 18, Max. = 42). For women, the mean age was 23.13 years (SD = 4.92, Min. = 18, Max. = 39). The mean length of the relationship was 42.78 months (SD = 44.02, Min. = 6, Max. = 204). From the entire sample, 15 couples were married. At least one partner from each couple was enrolled in a Psychology course at a Romanian University. Both their and their partner's participation was voluntary. For their participation, the participants received credits for their course.

### Procedure

Each participant received an online form containing the informed consent, the Sense of Relational Entitlement scale, and some demographic questions. In addition, they were asked to offer their email address and phone number. After sending back this information, each participant received another unique online form (containing an open-ended question regarding the most important topic of conversation for that day and the items measuring PPR, self-disclosure, perceived partner self-disclosure, and couple satisfaction), especially designed for him/her. They were asked to complete it twice a day (once at noon and once in the evening) for 7 days, from Monday to Sunday. Each day, one of the researchers sent personalized emails and phone messages to the participants in order to emphasize the importance of their adherence to the research. The protocol for this study was approved by the ethical committee of the university.

### Measures

#### The Sense of Relational Entitlement

We used the Romanian version of the Sense of Relational Entitlement scale (Candel, 2018). This scale contains 18 items that assess each person's relational entitlement type. The scale offers different scores for excessive (eight items), restricted (three items), and assertive entitlement (seven items; it includes both assertive and expectation items, as recommended by George-Levi et al., 2014). The items are rated on a Likert scale ranging from 1 (not at all) to 7 (very much). Each measurement offered good internal consistency, for both male and female participants (for restricted entitlement: Cronbach's α = 0.69 for males and 0.71 for females; for excessive entitlement: Cronbach's α = 0.86 for males and 0.87 for females; for assertive entitlement: Cronbach's α = 0.70 for males and 0.73 for females).

#### Couple Satisfaction

The participant's couple satisfaction was assessed using a single item (“Today I am satisfied with my relationship”) rated on a Likert scale ranging from 1 (total disagreement) to 6 (total agreement).

The other variables were measured using a single item: self-disclosure (“How much did you self-disclose since the last answers?”), perceived partner self-disclosure (“How much did your partner self-disclose since the last answers?”), and PPR (“How responsive was your partner since the last answers?”). All three items were measured on a Likert scale form 1 (not at all) to 6 (very much).

### Data Analytic Approach

The analysis used a total of 2,772 units of observations (99 couples × 2 members × 14 assessments). We analyzed these data using a multilevel model for dyadic diary data that treats the three levels of distinguishable dyadic diary data (days, nested within persons, nested within couples) as two levels of random variation. This method estimated separate intercepts and slopes for the male and female partner. The lower level represents variability due to day-level repeated measures for male partners and female partners, and the upper level represents person-level variability across male partners and across female partners (Bolger and Laurenceau, 2013). For each type of SRE, which were separately considered as upper-level predictors, we were interested in the person-level effects (e.g., the degree to which a person was characterized by greater excessive SRE at the beginning of the diary). For the moderators, we were interested in both person-level effects (e.g., the degree to which a person was characterized by greater self-disclosure over the course of the diary) and day-level effects (e.g., the degree to which a certain day was characterized by greater self-disclosure than the person's average). For this reason, we tested a model in which couple satisfaction was predicted by each type of SRE, the participants' averages of self-disclosure, perceived partner, and PPR, alongside daily deviations from these averages. In addition, we tested the interaction of each type of SRE with both the day-level moderators (cross-level interactions) and person-level averages of the moderators (level-2/person-level interaction).

Satisfaction_ijk_ = (male)_i_[γ_10_Self-disclosure_ijk_ + γ_20_Partner self-disclosure_ijk_ + γ_30_PPR_ijk_ + γ_01_Self-disclosure_ij_ + γ_02_Partner self-disclosure_ij_ + γ_03_PPR_ij_ + γ_04_Excessive SRE_ij_ + γ_05_Restricted SRE_ij_ + γ_06_Assertive SRE_ij_ + u_m0i_ + u_m1i_Self-disclosure_ijk_ + u_m2i_Partner self-disclosure_ijk_ + u_m3i_PPR_ijk_ + γ_07_(Self-disclosure_ij_ Excessive SRE_ij_) + γ_08_(Self-disclosure_ij_ Restricted SRE_ij_) + γ_09_(Self-disclosure_ij_ Assertive SRE_ij_) + γ_010_(Partner self-disclosure_ij_ Excessive SRE_ij_) + γ_011_(Partner self-disclosure_ij_ Restricted SRE_ij_) + γ_012_(Partner self-disclosure_ij_ Assertive SRE_ij_) + γ_013_(PPR_ij_ Excessive SRE_ij_) + γ_014_(PPR_ij_ Restricted SRE_ij_) + γ_015_(PPR_ij_ Assertive SRE_ij_) + γ_11_(Self-disclosure_ijk_ Excessive SRE_ij_) + γ_12_(Self-disclosure_ijk_ Restricted SRE_ij_) + γ_13_(Self-disclosure_ijk_ Assertive SRE_ij_) + γ_21_(Partner self-disclosure_ijk_ Excessive SRE_ij_) + γ_22_(Partner self-disclosure_ijk_ Restricted SRE_ij_) + γ_23_(Partner self-disclosure_ijk_ Assertive SRE_ij_) + γ_31_(PPR_ijk_ Excessive SRE_ij_) + γ_32_(PPR_ij_ Restricted SRE_ij_) + γ_33_(PPR_ijk_ Assertive SRE_ij_)] + (female)_1_[γ_40_Self-disclosure_ijk_ + γ_50_Partner self-disclosure_ijk_ + γ_60_PPR_ijk_ + γ_016_Self-disclosure_ij_ + γ_017_Partner self-disclosure_ij_ + γ_018_PPR_ij+_ γ_019_Excessive SRE_ij_ + γ_020_Restricted SRE_ij_ + γ_021_Assertive SRE_ij_ + u_f0i_ + u_f1i_ Self-disclosure_ijk_ + u_f2i_ Partner self-disclosure_ijk_ + u_f3i_ PPR_ijk_ + γ_022_(Self-disclosure_ij_ Excessive SRE_ij_) + γ_023_(Self-disclosure_ij_ Restricted SRE_ij_) + γ_024_(Self-disclosure_ij_ Assertive SRE_ij_) + γ_025_(Partner self-disclosure_ij_ Excessive SRE_ij_) + γ_026_(Partner self-disclosure_ij_ Restricted SRE_ij_) + γ_027_(Partner self-disclosure_ij_ Assertive SRE_ij_) + γ_028_(PPR_ij_ Excessive SRE_ij_) + γ_029_(PPR_ij_ Restricted SRE_ij_ + γ_030_(PPR_ij_ Assertive SRE_ij_) + γ_41_(Self-disclosure_ijk_ Excessive SRE_ij_) + γ_42_(Self-disclosure_ijk_ Restricted SRE_ij_) + γ_43_(Self-disclosure_ijk_ Assertive SRE_ij_) + γ_51_(Partner self-disclosure_ijk_ Excessive SRE_ij_) + γ_52_(Partner self-disclosure_ijk_ Restricted SRE_ij_) + γ_53_(Partner self-disclosure_ijk_ Assertive SRE_ij_) + γ_61_(PPR_ijk_ Excessive SRE_ij_) + γ_62_(PPR_ijk_ Restricted SRE_ij_) + γ_63_(PPR_ijk_ Assertive SRE_ij_)] + e_ijk_

In this double intercept model, Satisfaction_ijk_ is the predicted couple satisfaction for participant *i* in couple *j* on day *k*; male_i_ and female_i_ represent each gender's intercepts. On the day level, we introduced as predictors the daily levels of self-disclosure, perceived partner self-disclosure, and PPR for participant *i* in couple *j* on day *k*. At this level, γ _10_ and γ_40_ represent self-disclosure, _γ20_ and γ_50_ represent perceived partner self-disclosure, and γ_30_ and γ_60_ represent PPR. On the person level, we introduced as predictors the overall levels of excessive SRE, restricted SRE, assertive SRE, self-disclosure, perceived partner self-disclosure, and PPR for participant *i* in couple *j*. At this level, γ_01_ and γ_016_ represent self-disclosure, γ_02_ and γ_017_ represent perceived partner self-disclosure, γ_03_ and _γ018_ represent PPR, γ_04_ and γ_019_ represent excessive SRE, γ_05_ and γ_020_ represent restricted SRE, and γ_06_ and γ_21_ represent assertive SRE. u_m0i_ and u_f0i_ represent random intercepts, u_m1i_ and u_f1i_ represent random slopes for self-disclosure, u_m2i_ and u_f2i_ represent random slopes for perceived partner self-disclosure, and u_m3i_ and u_f3i_ represent random slopes for PPR. e_ijk_ is a residual component for this subject on the particular day. Additionally, this model also included person-level interactions and cross-level interactions between the SRE types and self-disclosure, perceived partner self-disclosure, and PPR for men and women, respectively.

All level-1 predictors were group-mean centered. All day-level effects were considered random and thus were allowed to vary from person to person. Each level-2 predictor was grand mean centered. All the analyses were computed using the IBM SPSS 20 software. To explore the interaction slopes, we estimated simple slopes for low (−1 SD), average, and high (+1 SD) levels of the moderators using the Preacher et al. (2006) computational tool for testing interaction effects in multilevel analysis.

## Results

### Preliminary Analysis

Means, standard deviations, and the paired-sample *t*-tests for the gender differences of each of the studied variables are presented in Table 1. Women report higher daily self-disclosure and assertive SRE. Men report higher levels of daily couple satisfaction. Table 2 presents the correlations between the variables. Couple satisfaction is positively related to self-disclosure, perceived partner self-disclosure, and PPR at both levels of the analysis. SRE is unrelated to the person-level self-disclosure, perceived partner self-disclosure, and PPR and negatively related to couple satisfaction.

**Table 1:** Descriptive statistics and gender differences for the variables.

	**Men**		**Women**		**Gender differences**
	**M**	**SD**	**M**	**SD**	**t**
Self-disclosure	5.05	1.24	5.18	1.20	−2.88**
Perceived partner Self-disclosure	4.95	1.27	5.00	1.25	−1.32
Perceived partner responsiveness (PPR)	4.88	1.25	4.83	1.35	1.21
Couple satisfaction	5.46	0.92	5.35	1.01	3.49***
Excessive sense or relational entitlement (SRE)	2.22	1.13	2.39	1.14	−1.16
Restricted SRE	3.12	1.54	2.81	1.51	1.38
Assertive SRE	4.53	0.99	4.79	0.94	−2.06*

* p < 0.05;

** p < 0.01;

*** *p < 0.001; the results are based on the day-level measurements for all level 1 variables and on the person-level measurement for SRE*.

**Table 2:** Correlations between the variables.

	**Couple satisfaction**	**Self-disclosure**	**Perceived partner self-disclosure**	**PPR**	**Excessive SRE**	**Restricted SRE**	**Assertive SRE**
Couple satisfaction	0.28***	0.41***	0.48***	0.48***	−0.51***	−0.07	−0.13
Self-disclosure	0.13***	0.18***	0.87***	0.62***	−0.14*	0.05	0.124
Perceived partner self-disclosure	0.18***	0.42***	0.21***	0.73***	−0.19***	0.08	0.11
PPR	0.21***	0.33***	0.54***	0.210***	−0.21**	0.11	0.02
Excessive SRE					0.23*	0.26***	0.46***
Restricted SRE						***−0.01***	0.125
Assertive SRE							***0.16***

* p < 0.05;

** p < 0.01;

*** *p < 0.001. Person-level correlations are presented above the diagonal and were calculated by averaging the daily responses over the entire diary period for each participant (N = 198). Day-level correlations are presented below the diagonal and were calculated using person-mean centered variables measured twice a day. On the diagonal, we included the correlations between men's and women's values (presented in bold and italic). For this, we used the day-level scores for couple satisfaction, self-disclosure, perceived partner self-disclosure and PPR, and the person-level scores for excessive, restricted, and assertive SRE*.

### The Person-Level Effects on Couple Satisfaction

The results of the hierarchical linear models regarding couple satisfaction are presented in Table 3. At person-level, excessive entitlement is significantly associated with couple satisfaction, meaning that the participants with greater excessive entitlement also report lower couple satisfaction. These associations are significant for both men and women (men: b = −0.25, SE = 0.05, *p* < 0.001; women: b = −0.28, SE = 0.06, *p* < 0.001). Restricted and assertive SRE were not related to couple satisfaction. The PPR was associated with greater couple satisfaction for men (b = 0.28, SE = 0.09, *p* < 0.001) and women (b = 0.28, SE = 0.09, *p* = 0.003). This indicates that when the participants perceived greater partner responsiveness over the course of the diary, they also reported higher couple satisfaction. Self-disclosure was associated with satisfaction for men (b = 0.24, SE = 0.10, *p* = 0.03), meaning that men that self-disclose more have, in general, higher levels of couple satisfaction.

**Table 3:** Self-disclosure, perceived partner self-disclosure, perceived partner responsiveness (PPR), and sense of relational entitlement (SRE), as predictors of couple satisfaction.

	**Men**				**Women**			
	**Estimate**	**SE**	**95% CI**		**Estimate**	**SE**	**95% CI**	
Intercept	5.42***	0.05	5.32	5.52	5.35***	0.06	5.24	5.47
*Day Level*								
Self-disclosure	0.04	0.03	−0.03	0.10	0.05	0.03	−0.02	0.10
Partner self-disclosure	0.04	0.03	−0.02	0.10	0.06	0.03	−0.01	0.13
PPR	0.13***	0.03	0.06	0.19	0.15***	0.03	0.07	0.22
*Person Level*								
Excessive SRE	−0.25***	0.05	−0.36	−0.15	−0.28***	0.06	−0.39	−0.16
Restricted SRE	0.003	0.03	−0.06	0.06	−0.03	0.03	−0.10	0.03
Assertive SRE	−0.06	0.06	−0.17	0.05	0.06	0.06	−0.07	0.18
Self-disclosure	0.24*	0.10	0.02	0.46	0.20	0.13	−0.06	0.48
Partner Self-disclosure	−0.02	0.12	−0.28	0.23	−0.05	0.16	−0.37	0.26
PPR	0.28***	0.08	0.11	0.45	0.28**	0.09	0.08	0.48
Self-disclosure × Excessive SRE	−0.27*	0.11	−0.51	−0.04	−0.13	13	−0.41	0.14
Self-disclosure × Restrictive SRE	−0.02	0.2	−0.07	0.01	−0.22*	0.10	−0.43	−0.01
Self-disclosure × Assertive SRE	0.05	0.04	−0.03	0.14	0.46**	0.16	0.13	0.79
Partner Self-disclosure × Restrictive SRE	0.19*	0.08	0.01	0.36	−0.07	0.11	−0.15	0.30
Partner Self-disclosure × Assertive SRE	0.06	0.16	−0.27	0.21	−0.54**	0.18	−0.90	−0.18
PPR × Excessive SRE	0.51***	0.09	0.33	0.70	0.13	0.06	−0.01	0.25
PPR × Restrictive SRE	−0.19**	0.06	−0.33	−0.06	0.13	0.06	0.01	0.26

* p < 0.05;

** p < 0.01;

*** *p < 0.001*.

### The Day-Level Effects on Couple Satisfaction

The day-level analysis yielded fewer significant results. Only PPR significantly predicted couple satisfaction, for both men and women (for men: b = 0.13, SE = 0.03, *p* < 0.001; for women: b = 0.15, SE = 0.03, *p* < 0.001). In the days when men and women perceived greater partner responsiveness, they reported higher satisfaction with their relationship. No other result was significant.

### The Moderation Effect of Self-Disclosure, Perceived Partner Disclosure, and Perceived Partner Responsiveness

No cross-level interactions were found. However, several person-level interactions were significant. The plots for all the significant interactions are included as Supplementary Material. The relationship between excessive SRE and couple satisfaction is moderated by self-disclosure and PPR. These effects were significant only for men. Estimation of simple slopes indicate that men's higher excessive SRE is not associated with couple satisfaction at low levels of self-disclosure (b = −0.03, SE = 0.13, *p* = 0.80), but is significantly associated with lower couple satisfaction at medium (b = −0.25, SE = 0.09, *p* < 0.01) and high levels of self-disclosure (b = −0.48, SE = 0.13, *p* < 0.001). Also, men's higher excessive SRE is associated with lower couple satisfaction at low (b = −0.69, SE = 0.14, *p* < 0.001) and medium levels of PPR (b = −0.25, SE = 0.09, *p* < 0.01). However, it is not associated with couple satisfaction at high levels of PPR (b = 0.18, SE = 0.13, *p* = 0.19).

The link between restricted SRE and couple satisfaction was moderated by perceived partner disclosure and PPR (for men only) and by self-disclosure (for women only). For men, the relationship between restricted SRE and couple satisfaction is not significant at low (b = −0.15, SE = 0.10, *p* = 0.13) and medium levels of perceived partner disclosure (b = 0.003, SE = 0.07, *p* = 0.96), but becomes barely significant and positive at high levels of perceived partner disclosure (b = 0.16, SE = 0.09, *p* = 0.07). In addition, the relationship between restricted SRE and couple satisfaction is not significant at low (b = 0.17, SE = 0.10, *p* = 0.11) and medium levels of PPR (b = 0.003, SE = 0.06, *p* = 0.96), but becomes barely significant and negative at high levels of PPR (b = −0.16, SE = 0.09, *p* = 0.08). Finally, at low (b = 0.14, SE = 0.13, *p* = 0.28) and medium levels of self-disclosure (b = 0–0.03, SE = 0.07, *p* = 0.63), women's level of restricted SRE is not related to couple satisfaction. However, the relationship becomes significant and negative at high levels of self-disclosure (b = −0.21, SE = 0.10, *p* = 0.04).

Self-disclosure and perceived partner disclosure moderate the relationship between assertive SRE and couple satisfaction (for women only). Assertive SRE has a barely significant negative association with couple satisfaction at low levels of self-disclosure (b = −0.30, SE = 0.18, *p* = 0.07). This relationship becomes non-significant at medium levels of self-disclosure (b = 0.05, SE = 0.11, *p* = 0.62). At high levels of self-disclosure, the association is significant and positive (b = 0.44, SE = 0.14, *p* < 0.01). At low levels of perceived partner disclosure, there is a significant and positive association between assertive SRE and couple satisfaction (b = 0.51, SE = 0.17, *p* ≤ 0.01). This association becomes non-significant at medium levels of perceived partner disclosure (b = −0.05, SE = 0.11, *p* = 0.62), and significant and negative at high levels of partner disclosure (b = −0.40, SE = 0.14, *p* ≤ 0.01).

## Discussions

Past research has shown that people that are either excessively or restrictedly entitled use maladaptive ways of need expression and that they may report lower levels of couple satisfaction (Tolmacz and Mikulincer, 2011; George-Levi et al., 2014). Besides, disclosing information about the self and about the current needs toward the romantic partner and the way the partner responds to this can affect couple satisfaction (Sprecher and Hendrick, 2004; Canevello and Crocker, 2010; Unger et al., 2015). Using dyadic diary data from romantic couples, the current study explored the possibility that self-disclosure, perceived partner self-disclosure, and PPR would moderate the relationships between excessive, restrictive, and assertive SRE and couple satisfaction.

The first hypothesis, regarding the relationship between SRE and couple satisfaction, was only partially supported. People with higher levels of excessive SRE reported lower levels of couple satisfaction. However, contrary to previous results (Tolmacz and Mikulincer, 2011), restricted SRE was not related to couple satisfaction. In another study on the same model of the SRE, George-Levi et al. (2014) suggested that excessive and restricted entitlement should be grouped in one new factor called conflicted entitlement. As such, these two types of entitlement may share some variance when it comes to explaining the variation in couple satisfaction. Given that excessive entitlement was previously shown to have a stronger relationship with couple satisfaction (George-Levi et al., 2014), this may account for the non-significant association between restricted SRE and couple satisfaction. Finally, assertive SRE was not related to couple satisfaction, a finding that confirms previous studies (Tolmacz and Mikulincer, 2011).

We proposed that self-disclosure is related to couple satisfaction. This hypothesis was only partially supported. Men's self-disclosure is related to their couple satisfaction, but only at the personal level. Day-to-day self-disclosure does not seem to be related to daily levels of couple satisfaction, a finding that contradicts some previous results (e.g., Rosenfeld and Bowen, 1991). These findings may be explained by the fact that self-disclosure, although it has some aspects of personality construct, is also greatly influenced by relational or environmental conditions (Sprecher and Hendrick, 2004). As such, the relationship between daily self-disclosure and daily couple satisfaction might be affected by other variables. Also, emotional self-disclosure seems to be more important than factual self-disclosure (Laurenceau et al., 2005), but in this study, we did not differentiate between the two. As for the gender differences, previous studies (Dindia and Allen, 1992) have shown that women disclose more than men, and the present results confirm these findings. However, only men's higher general levels of self-disclosure are associated with higher levels of couple satisfaction. Laurenceau et al. (2005) found that self-disclosure is more important for a male than it is for women in predicting intimacy. Although not identical, the process regarding couple satisfaction can be similar. Male partners can be more reliant on engaging in self-revealing disclosure, while female partners may derive their couple satisfaction from other components of the process (such as PPR).

The third hypothesis stated that perceived-partner self-disclosure is associated with couple satisfaction. We found no significant association at any level and for either gender. Although surprising, this may be explained by people's overestimation of their partner's disclosure. Rosenfeld and Bowen (1991) state that people have a tendency to consider their partner's self-disclosure similar to their own. These present results show a very strong correlation between self-disclosure and perceived partner self-disclosure, a fact that supports this assumption. As such, partner disclosure might act in a very similar way to self-disclosure.

The fourth hypothesis was confirmed. Perceived Partner Responsiveness was significantly related to higher couple satisfaction for all the participants, at both day and person levels. These findings support the previous results (Bar-Kalifa et al., 2015; Segal and Fraley, 2016) and show that feelings of understanding, validation, and acceptance from the partner are extremely important in shaping one's couple satisfaction toward the relationship.

The hypothesis concerning the moderating role of self-disclosure, perceived partner self-disclosure, and PPR was only partially supported. First, a significant association between higher excessive SRE and lower couple satisfaction was observed only for the men that used more self-disclosure. Although contrasting with the proposed hypothesis, this result finds its support in the studies showing the negative effects of too much self-disclosure (Cozby, 1972; Collins and Miller, 1994). High levels of self-disclosure can leave the recipient unsure of how to respond, leading to their constant retreat from the relationship. This might be particularly damaging for an excessively entitled person who might not easily forgive such a transgression, feeling that their personal needs are not fulfilled by the partner. Moreover, entitled individuals use various self-promotion behaviors, self-disclosure being one of them, but are also unethical in their decision-making style (Tamborski et al., 2012; Abell and Brewer, 2014). They can promote their needs in the relationship with their partner by self-disclosing, but might not reciprocate when the partners also express their needs. Thus, the partners can distance themselves from the entitled individuals, with the latter becoming less satisfied. Finally, people can also self-disclose their negative feelings, which might lead to negative reciprocity from the partner and further dissatisfaction (Finkenauer et al., 2018). Excessively entitled individuals might be more prone to self-disclose their disappointments following their partner's transgressions. When the level of their negative self-disclosure gets stronger, their satisfaction might become weaker. On the contrary, perceiving one's partner as being more responsive is beneficial for the more entitled individuals. In agreement with our hypothesis, perceived partner responsiveness buffers the negative effect of excessive SRE on couple satisfaction. Having a partner that is more sensible and responsive toward one's needs was found to be related to higher couple satisfaction (Gadassi et al., 2016). This seems to play an important role in determining someone with strong unmet emotional needs to feel more satisfied. Feeling that the partner is caring and understanding is beneficial for men with higher levels of excessive entitlement. Finally, perceived partner responsiveness might also appear due to the individual's own projection of responsiveness (Lemay et al., 2007). Due to an underlying narcissism, those with an excessive entitlement can consider themselves as being more responsive to their partners' needs. Thus, they might maintain the perception of a responsive partner and their relational satisfaction due to their personality traits.

The moderator analysis for the relationship between restricted entitlement and couple satisfaction provided some contradictory findings. First, the link is negative only at high levels of either PPR or self-disclosure (the former moderator was significant for men, while the latter was significant for women). Restricted entitlement consists of the belief that one does not deserve to get anything from the partner. However, both high PPR and high self-disclosure foster intimacy, a process where the partners listen to each other and are attentive to one another (Prager, 1995). This might contradict the core beliefs of inadequacy that a restrictively entitled person possesses, leading to confusion, guilt, shame, and low couple satisfaction. Paradoxically, higher levels of restricted SRE and higher levels of perceived partner self-disclosure interact and predict higher levels of couple satisfaction for men. Tolmacz (2011) proposes that a restricted sense of entitlement can emerge from maternal messages that communicate dissatisfaction with the child. Therefore, the individual starts believing in their usefulness. Later, the adult would act in such a way to satisfy the partner to compensate for their perceived ineptness. Our results suggest that partner disclosure offers the ideal opportunity for individuals with a restricted entitlement to feel useful. Specifically, by allowing their partners to disclose, they consider that they atone for their past unfitness, which makes them feel more satisfied. Nevertheless, the positive impact of perceived partner self-disclosure might also be explained by the capitalization theory (Langston, 1994). Self-disclosing about positive events can lead to more trust toward the target person (Reis et al., 2010). Although we measured just the perception of partner self-disclosure and not the actual disclosure, it is possible for them to be positively correlated. This means that the partners after they self-disclose, offer more trust to the restrictive entitlement individuals, which might determine the latter to capitalize on these positive experiences. In the end, it is possible for the individuals with a restricted sense of entitlement to capitalize more from their partners' positive experiences than from their own.

Assertive entitlement is significantly and positively related to couple satisfaction only when the person discloses more. This result was found only among women. For assertively entitled individuals it is important to obtain what they feel they deserve. This attitude, combined with a higher ability to self-disclose and communicate in a non-aggressive way about their needs, can determine the partner to pay more attention to the person's needs. They might also capitalize on their positive self-disclosure and increase their satisfaction by talking about their positive experiences. Moreover, when low levels of self-disclosure are achieved, the assertive persons lack one important mechanism used to express their needs. Therefore, they seem to be less satisfied. On the contrary, the level of perceived partner disclosure intensifies dissatisfaction among assertively entitled women. This shows that while assertive women need to disclose more to be more satisfied, they do not want to reciprocate and allow the partners to disclose. Previous studies showed that assertive entitlement was positively related to some facets of narcissism, such as superiority and vanity (Tolmacz and Mikulincer, 2011). Despite being the more adaptive type of entitlement, assertive entitlement still overlaps with some narcissistic traits. Also, taking into account the results of Crowe et al. (2016) and Hart et al. (2019), assertive entitlement can be described as a more emotionally stable and less vulnerable form of entitlement, but not completely devoided of the antagonistic behavior that can be found in maladaptive entitlement. As such, when faced with greater partner disclosure and greater expression of needs form their partner, the individuals with greater assertive entitlement might not feel prepared to respond and thus might report less couple satisfaction.

Significant gender differences emerged during the analysis. Self-disclosure, perceived partner disclosure, and PPR mostly played different roles among men and women and interacted differently with the facets of SRE. One potential explanation for this is that there are gender differences in the process of intimacy. Women generally self-disclose more than men (Dindia and Allen, 1992; Horne and Johnson, 2018), and this result was supported by our findings. Moreover, other studies suggest that women feel more satisfied with the process of intimacy when the partner self-discloses more, while for men, both partners must disclose (Manne et al., 2004). Moreover, women tend to respond with better accuracy to their partner's expression of needs, being responsive when the partners experience greater stress. On the contrary, men offer both responsiveness and negative behaviors when the partner needs support (Neff and Karney, 2005).

As a summary, this study shows that the components of the interpersonal process of intimacy can both buffer and aggravate the effects of SRE on couple satisfaction. For excessively entitled individuals, offering more self-disclosure seems to be counterproductive. However, having a more responsive partner allows for greater couple satisfaction. Still, this positive effect of PPR can be only temporary, depending on the ability and willingness of the partner to be responsive toward partners who greatly exaggerate their needs and concentrate mostly on themselves. It is worth noting that these results were found only in males. As previously mentioned, women are generally better at responding to their partner's moments of greater stress. Thus, excessively entitled men might risk taking this ability for granted. For restricted individuals, higher intimacy promoting behaviors (higher self-disclosure and PPR) may contrast with their low or non-existent expectations, bringing a decline in their satisfaction. On the contrary, greater perceived partner disclosure may come with the opportunity to feel useful and increase their couple satisfaction. In the end, assertive entitled individuals profit from greater self-disclosure and report more couple satisfaction, but seem to be affected by greater partner self-disclosure. Although the least damaging form on entitlement, assertiveness can also bring negative consequences when the partners insist too much on their needs. Alternatively, given that this result was found only on women, assertive women can achieve lower couple satisfaction when their partner discloses more because self-disclosure is not a behavior that fits with the gender role expected from men. Our results support the view of Finkel et al. (2017) on the role of responsiveness in romantic relationships. While being responsive promotes couple satisfaction, this is highly dependent on the individual's predispositions. In conjunction with self-disclosure and PPR, the different types of entitlement lead to different levels of couple satisfaction.

In addition to theoretical advances, this study also proposes some methodological strengths. To our knowledge, this is one of the first studies that used the concept of SRE and all three of its forms in a diary design. This kind of research is very useful because it allows the study of the participants in a more ecologically valid way, partially suppressing the shortcomings of a cross-sectional design. Also, while previous studies that investigated this concept concentrated in more experienced couples, our results point to some similar findings in a sample of young couples.

However, this study is not without its limits. While the diary design allows for a long investigation, it remains correlational, and thus, it does not allow for inferring a causal association between the variables. Also, all the concepts were measured with self-reporting questionnaires and the sample was mostly composed of couples with a relatively high socioeconomic status that presents higher than average couple satisfaction. In the future, other methods (such as direct observation) and other samples can be used to extend these results. Finally, although entitlement is distinct from narcissism, the two concepts are related. Future studies should control the role of narcissism to explore how SRE affects couple satisfaction above and beyond it.

## Conclusion

This present study examined the moderation effect of self-disclosure and PPR on the associations between SRE and couple satisfaction. Our main findings indicate a negative association of daily couple satisfaction with excessive SRE, but not with restricted SRE. Self-disclosure was related to couple satisfaction, but only for men and only at person-level. Perceived-partner self-disclosure was related with couple satisfaction for men and women at both day-level and person-level. All three types of SRE (assertive, restricted, and excessive) interact with self-disclosure, perceived partner disclosure, and perceived partner responsiveness and account for changes in couple satisfaction. To our knowledge, this is the first study to use the variables included in the interpersonal process model of intimacy (Reis and Shaver, 1988) as an organizing construct for the interactions between SRE and couple satisfaction. The research on relational entitlement is recent, and only a few studies have examined its importance in shaping the levels of couple satisfaction. Still, our results are important, because other than their empirical strengths, they can point to some clinical and therapeutic implications too. For example, they suggest that careful expression of needs thought self-disclosure and a responsive answer from the partner can overcome the effects of some of the more damaging types of entitlement. Based on these results, the therapists would be able to create programs that take into account the level of excessive, restricted, and assertive relational entitlement when advising greater use of self-disclosure and PPR. Finally, these programs should use different techniques depending on gender.

## Data Availability Statement

The raw data supporting the conclusions of this article will be made available by the authors, without undue reservation.

## Ethics Statement

The studies involving human participants were reviewed and approved by Ethical Committee of the Alexandru Ioan Cuza University. The patients/participants provided their written informed consent to participate in this study.

## Author Contributions

OSC and MNT wrote the manuscript, conceived and designed the study. OSC collected and analyzed the data.

## Conflict of Interest

The authors declare that the research was conducted in the absence of any commercial or financial relationships that could be construed as a potential conflict of interest.
