# Supplementary material for: The Role of Relational Entitlement, Self-Disclosure and Perceived Partner Responsiveness in Predicting Couple Satisfaction: A Daily-Diary Study
Source: Front Psychol. 2021 Mar 23;12:609232. doi: 10.3389/fpsyg.2021.609232 (PMC8022838; doi:10.3389/fpsyg.2021.609232)

Supplementary Material: Interaction Plots for the Significant Person-level Interactions

1 Supplementary Figures

Supplementary Figure 1. *The moderating role of men's person-level self-disclosure in the association between men's excessive SRE and their daily couple satisfaction*

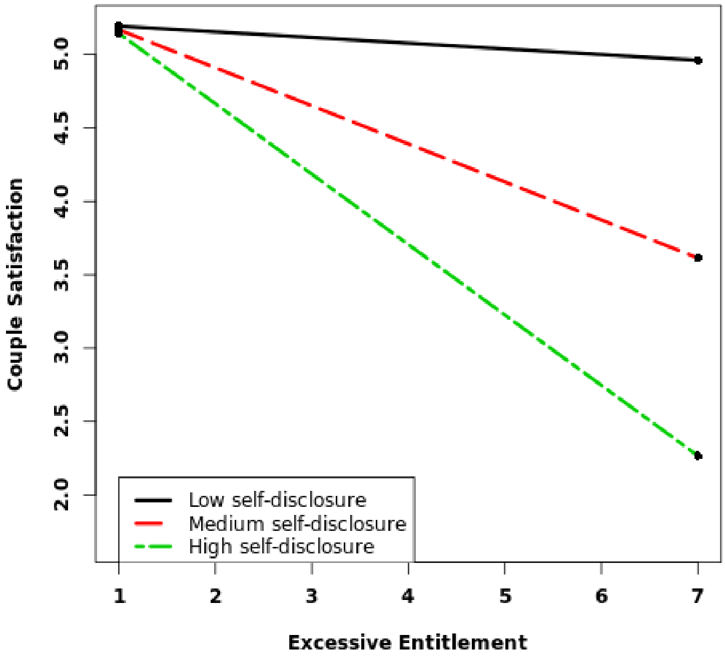

Supplementary Figure 2. *The moderating role of men's person-level perceived partner responsiveness in the association between men's excessive SRE and their daily couple satisfaction*

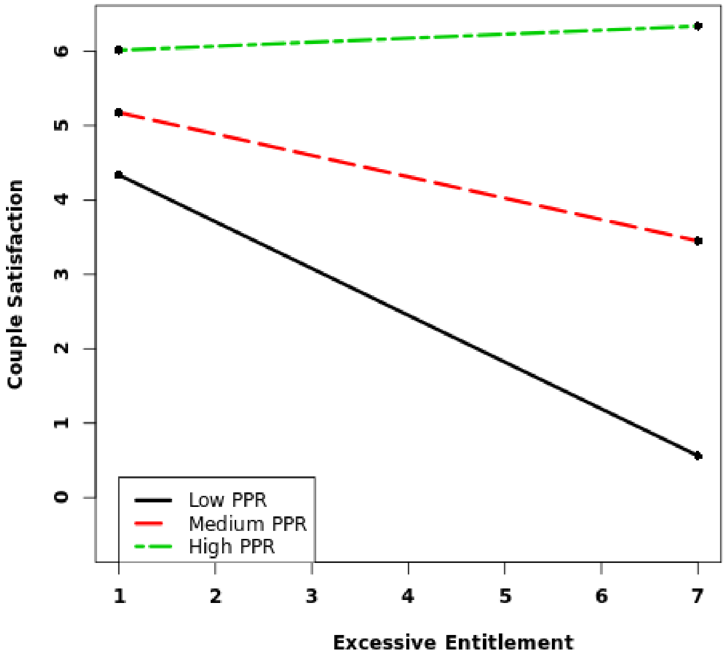

Supplementary Figure 3. *The moderating role of men's person-level perceived partner self-disclosure in the association between men's restricted SRE and their daily couple satisfaction*

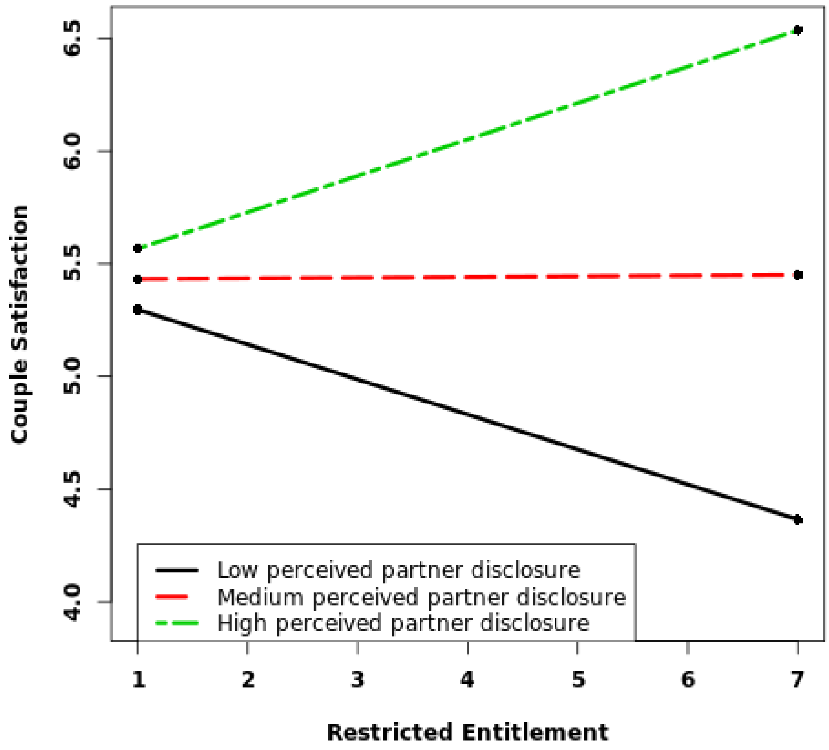

Supplementary Figure 4. *The moderating role of men's person-level perceived partner responsiveness in the association between men's restricted SRE and their daily couple satisfaction*

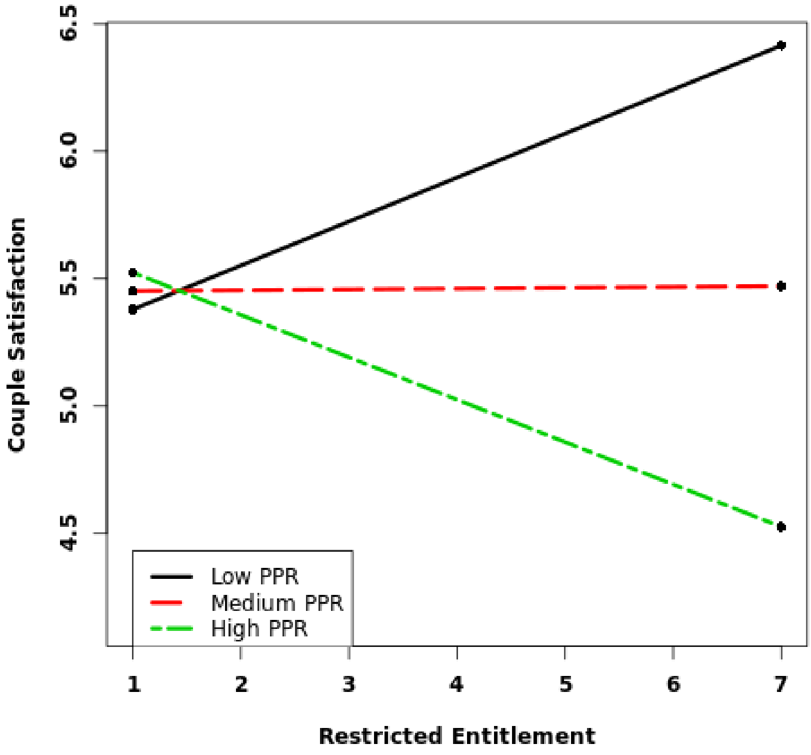

Supplementary Figure 5. *The moderating role of women's person-level self-disclosure in the association between women's restricted SRE and their daily couple satisfaction*

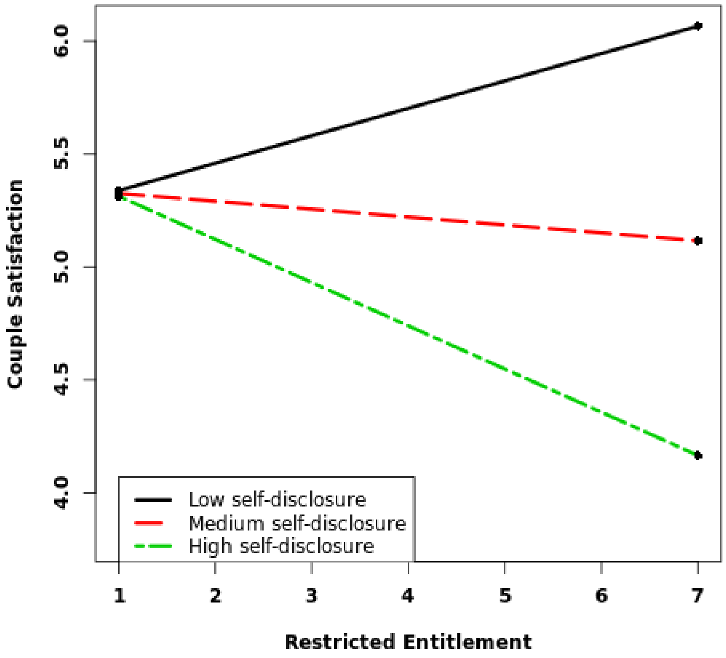

Supplementary Figure 6. *The moderating role of women's person-level self-disclosure in the association between women's assertive SRE and their daily couple satisfaction*

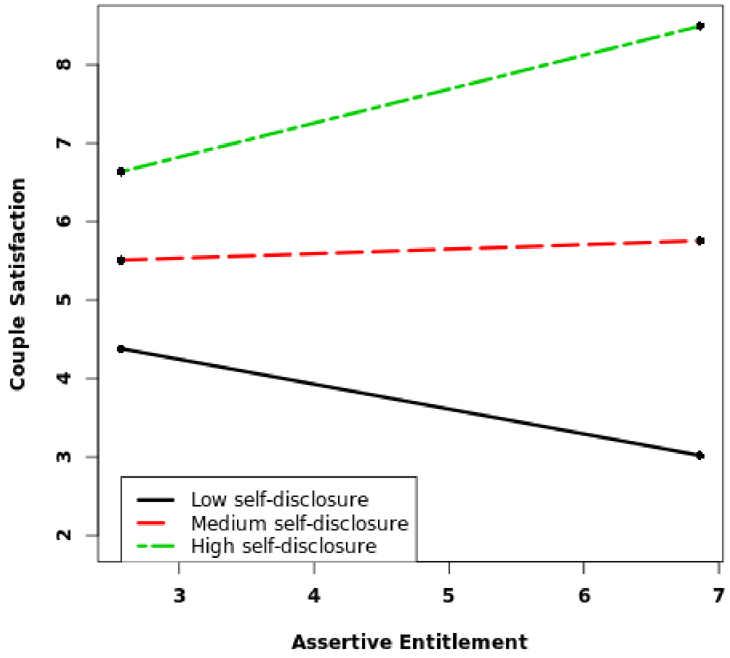

Supplementary Figure 7. *The moderating role of women's perceived partner self-disclosure in the association between women's assertive SRE and their daily couple satisfaction*

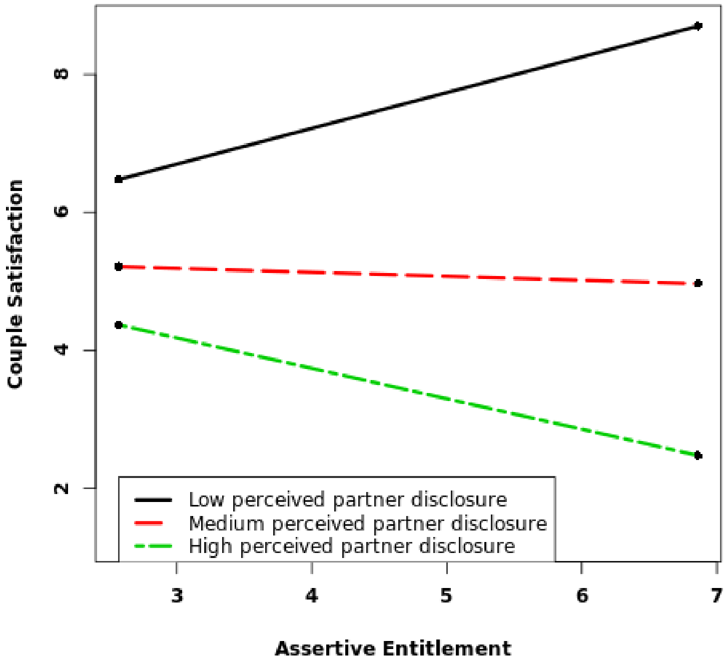

Supplement: Supplementary file 1 [file Data_Sheet_1.pdf]
